# Peer Victimisation in Early Childhood; Observations of Participant Roles and Sex Differences

**DOI:** 10.3390/ijerph18020415

**Published:** 2021-01-07

**Authors:** Claire P. Monks, Peter K. Smith, Kat Kucaba

**Affiliations:** 1 School of Human Sciences, Institute for Lifecourse Development, University of Greenwich, London SE10 9LS, UK; k.j.kucaba@gre.ac.uk; 2 Department of Psychology, Goldsmiths, University of London, London SE14 6NW, UK; p.smith@gold.ac.uk

**Keywords:** victimisation, aggression, early childhood, observations

## Abstract

During middle childhood and adolescence, victimisation appears to be a group process involving different participant roles. However, peer reports with younger children (four to six years old) have failed to identify the participant roles of assistant (to the bully) reinforcers or defenders with much reliability. This may be because peer victimisation is a more dyadic process among younger children (behavioural reality), or because of limitations in young children’s cognitive capacity to identify these behaviours (cognitive limitations). The findings of an observational study which examined the group nature of peer victimisation among young children are presented. Observations were made of 56 children aged four and five years using time sampling during free play at school (totalling 43.5 h of observation). Records were made of their behaviour when an onlooker witnessed aggression by others, and also of others’ behaviour when they were being aggressive or being victimised. Although children other than the aggressor and target were present in nearly two thirds of the episodes of peer victimisation observed, few exhibited behavioural responses in line with the assistant, reinforcer or defender roles. This supports the behavioural reality rather than the cognitive limitations explanation. Sex differences were observed in types of aggression displayed by children, with boys more likely than girls to be physically aggressive. Children were less likely to be aggressive to other-sex peers and were most likely to be victimised by children of the same sex as them. There were also sex differences in children’s onlooker behaviour. The implications for our understanding of the development of peer victimisation and bullying in children are discussed.

## 1. Introduction

There is a growing body of research on the nature and extent of peer victimisation among children during the preschool or early school years (between the ages of 4 and 6 years) [1,2,3,4,5,6]. Peer victimisation among such young children appears to differ from bullying as reported later in middle childhood and adolescence in several ways, including the stability and variety of roles taken [3]. It has been suggested that peer victimisation among younger children may not involve the wider peer group as has been found among older groups [7], with young children less likely to take roles of assisting or reinforcing the aggressor [8,9]. Understanding the roles that children take in peer victimisation is important for the development of appropriate intervention and prevention programmes, some of which have successfully addressed group dynamics within the peer group as a way of tackling bullying among older children [10,11,12,13,14,15]. However, there is still some debate over the group nature of peer victimisation among younger children, which may be related to the methodologies employed, in particular the common use of peer reports which may be affected by children’s capacity to understand and report on particular behaviours by their peers. The current study used observations of children aged four and five years during their first year in school, to examine the group nature of peer victimisation.

### 1.1. Peer-Directed Aggression and Sex Differences

Peer-directed aggression can take various forms, including physical (hitting and kicking), verbal (name calling and taunting) and relational (social exclusion), but among young children, it is most commonly direct, taking place in face-to-face encounters [16]. Research with older children indicates that boys are more likely to be aggressors than girls [17,18,19]. The types of aggression used by older groups also vary by sex, with boys more likely to use physical aggression, but there are no significant sex differences in relational aggression [20]. More recent research has confirmed that boys experience more physical and verbal aggression than girls [21].

Research with young children has indicated that when they behave aggressively, boys and girls are more likely to victimise children of their own sex [22,23]. Similarly to research with older children, research has indicated that boys are more likely than girls to be aggressors in younger groups [9], whereas girls are more likely than boys to defend victims [24,25], although being a victim does not show consistent sex differences [26,27].

### 1.2. The Role of the Peer Group in Victimisation

The importance of group processes as well as individual factors in understanding bullying in middle childhood and adolescence is now well established [7,28,29]. Observational studies have confirmed that bullying rarely occurs with only the aggressor/bully and victim present. Atlas and Pepler [30] conducted observations of children aged 6 to 12 years and found that peers were present during 85% of bullying episodes; peers actively participated in the bullying in 32% of episodes, were passive onlookers in 13% and intervened to stop the bullying in 10%. O’Connell et al. [31] also observed the behaviours of children during episodes of bullying, distinguishing three “onlooker” behaviours: peer actively joins in (“peer joins bully in physically or verbally abusing a victim”); peer passively joins in (“peer is clearly aware of bullying—onlooks for more than 5 s—but does not intervene or leave”); and peer intervenes (“peer offers support to a victim”) (p. 444). They found that boys aged over eight years were more likely than 5 to 8-year-olds to actively join in during episodes of bullying, and girls were more likely than boys to intervene during bullying. They observed “passive joining in” occurring 54% of the time in episodes of bullying and argued that this reinforced the aggressors’ behaviour as it provided them with an audience who did not intervene and thus may be viewed as silently condoning the bullying.

Salmivalli and colleagues [32] also indicated that “onlookers” were often actively or passively reinforcing the behaviour of the aggressor. They identified several participant roles in bullying: victim, ringleader bully (who starts the aggression), assistant (who helps the bully), reinforcer (who encourages the bullying), defender (who supports the victim), and outsider (who stays out of the bullying situation). There is some indication of stability of these roles over time during adolescence [33], although ethnographic approaches and retrospective interviews with victims indicate some fluidity in the roles [34,35]. Levels of bullying have been found to be related to the levels of reinforcing (positively) and defending (negatively) in classes of children aged 9–11 years, suggesting that bystander behaviour is important in affecting the levels of bullying by classmates [36]. Moreover, Salmivalli [37] indicated that changing the behaviour of bystanders may successfully diminish episodes of bullying in schools. The participant role approach involves changing the behaviour of bystanders and has been employed successfully in antibullying work among children aged 7 years and older [10,11,12,13,14,15].

There is evidence for all these participant roles being identifiable among children from the age of 7 years onwards [38,39]. However, the research findings among younger children have been less consistent.

Some studies have focussed on information from teachers of preschool children. In Italy, Belacchi and Farina [24,40], and Farina and Belacchi [41] asked teachers of 3–6-year-old children to identify eight roles in total (adding two new roles to the participant roles: consoler, someone who takes care of the victim, and mediator, someone who tries to negotiate within the bullying scenario). However, their analyses grouped these eight roles into four overarching roles of hostile, prosocial, victim, and outsider. Research in Finland did find that preschool teachers reported reinforcing and assisting behaviours being exhibited by children aged 3–6 years [42].

However, based on peer as well as teacher reports, Monks and colleagues [8,27] found that young children and teaching staff in England were unlikely to identify 4–6-year-olds as taking assistant and reinforcer roles. Furthermore, there was little agreement between peers and teachers about who exhibited these behaviours. By contrast, there was some consistency in reports by children and their teachers on 5-year-olds’ aggression, victimisation and defending [8]. Additionally, based on peer and teacher reports, Camodeca et al. [43], in research in Italy with 2–6-year-olds, collapsed reinforcing and assisting into a “follower” role. They found high intercorrelations between most of the roles, suggesting that these were not well distinguished.

In an observational study of 4- to 6-year-olds, Vlachou et al. [44] noted that many episodes of peer victimisation occurred where a number of children targeted one individual and that there were often other children present during episodes of peer victimisation. Although the majority of those present were passively observing what was happening, Vlachou et al. [44] did note that some were defending the victim or supporting the aggressor.

From the research to date, it appears that some preschool children are aggressive to others, and some defend others during episodes of peer victimisation. However, the less central roles of assistant and reinforcer may be less easily identified during early childhood. Two main hypotheses have been proposed to explain why fewer participant roles can be reliably found in groups of young children, and especially the assistant and reinforcer roles. One relates to behavioural reality, the other to cognitive limitations in reporting.

As regards behavioural reality, Monks et al. [26] proposed that the assistant, reinforcer and defender roles are indeed less often present among young children; peer victimisation may be less group-led. Children’s friendship groups are less stable during the early years at school and there is evidence that dominance hierarchies among groups of young children are more fluid and subject to change [45,46]. Therefore, they may be less likely to form aggressive subgroups than is observed among older children.

As regards cognitive limitations, it may be that young children have difficulty in identifying reinforcing or assisting behaviour among their peers, rather than these behaviours not being exhibited by particular children with any consistency. Young children find it difficult to identify withdrawn behaviours among their classmates, not because these behaviours do not occur, but perhaps because they are less salient and impactful on them [47,48]. This may be similar for assisting, reinforcing and defending behaviours, which may occur within this age group but not be identified by younger children due to their more limited cognitive capabilities. Furthermore, teaching staff may be less able to identify these more peripheral roles as they are often not present during episodes of peer victimisation [25], and these behaviours may be less likely to be reported to them by children.

Observations are an obvious way of examining whether these behaviours occur. The very few observational studies conducted on this topic with young children have either focussed on a limited range of victimisation behaviours [49] or have focussed on examining the group nature of peer victimisation at older ages [30,31]. Rose et al. [50] conducted observations of 108 aggressive incidents among 50 children aged 2–6 years in three preschool classes in the USA. They noted that other children were most often unaware of aggressive behaviours by peers, and seldom watched, reinforced the aggressor or defended the victim. However, their study included very young children aged 2–3 years, whose cognitions and behaviour may differ from those of 4- and 5-year-olds. To date, little research has examined the behaviours of onlookers in response to different forms of peer victimisation during early childhood. Research with older children and adolescents has indicated that young people’s responses to peer victimisation are influenced by a number of factors, including perceptions of the severity of the aggression [51]. Looking at defending behaviour in particular, there is evidence suggesting that individuals perceive physical aggression as more severe [52] and may thus be more likely to intervene when these types of aggression are demonstrated [51].

We conducted an observational study in order to overcome some of the issues related to peer reports from young children. We describe the forms of aggression seen, and sex differences in these; and the relative frequencies of cross-sex and same-sex aggression and victimisation were contrasted. The main aim of the study was to examine whether onlookers were present during episodes of peer victimisation and whether their behaviour reflected the participant roles of Salmivalli et al. [32], in particular, whether behaviour which was descriptive of reinforcing, assisting or defending was observed; this would discriminate between the behavioural reality or cognitive limitations explanations. Sex differences in onlooker behaviour were also examined, and the behaviour of onlookers was contrasted across different types of aggression and victimisation.

## 2. Materials and Methods

### 2.1. Participants

Two Reception classes of children (*N* = 56; aged 4 to 5 years; mean age = 62.11 months, *SD* = 3.36) from a school in South East England participated. Reception year is the first year of formal schooling of children in England. There were more boys than girls in the sample (male *N* = 35, 61.4%; female *N* = 22, 38.6%) which was an artefact of the distribution of boys and girls in the classes involved.

### 2.2. Observations

Continuous focal child (FC) observations were conducted using intervals of 10 min during children’s free-play activities (comprising indoor and outdoor activities) over a period of five weeks (with caveats that each child could only be observed once per day and the observation was terminated if the child was out of range for 30 s [53,54]). Free play comprised approximately 2–3 h of the day. Observations were noted using a field notebook, this method being chosen as less obtrusive than video or tape recordings and more acceptable to parents/carers and schools.

Behaviours were recorded whenever the FC was (a) behaving aggressively, (b) being victimised or (c) present when someone else was behaving aggressively or being victimised. In all such cases, the main type of aggression used was recorded as either: physical (hitting, kicking or pushing another child); verbal (shouting at someone or name calling); or relational (excluding or rumour spreading). In addition, if other children were present, or if the FC was present as in (c), their behaviour was noted, whether they were helping the aggressor to behave aggressively (joining in with the aggression); encouraging the aggressor (by laughing or cheering them on); helping the victim (by intervening or telling the aggressor to stop); or doing nothing [7,8,32].

Each behaviour was scored in terms of the number of times the child was observed exhibiting the behaviour, providing a “count” of the number of times the FC exhibited each behaviour during the observation interval. Incidents of involvement in aggression or observing aggression were coded as being separate incidents if there was at least a one-minute interval between the incidents. Therefore, it was possible that more than one incident of aggression could be recorded within a 10 min observation period. An individual (including the FC) was identified as being present during episodes of peer aggression or victimisation if they met the following criteria: they were in sufficiently close proximity to the incident (within five metres) and were observed looking at what was happening.

Two observers, both female, were trained on the observation schedule. Time was spent with each class prior to data collection to help the children to become accustomed to the observer being present [55] and to ensure accurate and speedy identification of children during the observation period. The observers observed different focal children at each time.

In total, 261 observations of 10 min duration were conducted (totalling 43.5 h of observation). A total of 38 children were observed five times, 17 were observed four times and 1 was observed three times during the five-week period. All observation scores were transformed into proportion scores to take into account this difference in the number of observations. Inter-rater reliability was calculated for 22 observations (totalling 220 min). This was carried out around the midpoint of the data collection period. Cohen’s κ was performed to examine inter-rater reliability across each of the observation categories. Agreement was significant; all kappas *p* < 0.001. Kappas ranged from 0.680, *p* = 0.001 (91.81% agreement) for observations of others ignoring the aggression/victimisation of the FC to 0.882, *p* = 0.001 (89.09% agreement) for the type of aggression observed by the FC. Where there were disagreements, these were discussed, and agreement was met.

### 2.3. Procedure

Ethical approval for the study was obtained from the relevant University Research Ethics Committee (ethics code: 11/12.3.5.16). Consent was obtained from headteachers and parents/guardians of participating children. The observers spent time with the classes prior to the observation period beginning so that the children were accustomed to their presence. Observations were conducted in the outside in the playground and within the classroom during free-play activities over several weeks.

## 3. Results

Of the 261 observations made, aggression was noted as occurring in 177 (67.8%), either with the FC as an aggressor, victim or observer. In total, 376 incidents of aggression were observed; however, for 6 incidents, the type of aggression was not recorded, so the nature of 370 incidents is shown in Table 1, and these are the episodes analysed below. The FC was most often observed as the aggressor (*N* = 150, 40.5%), followed by as an observer (*N* = 114, 30.8%) and victim (*N* = 106, 28.7%), *χ^2^* (2*df*, *N* = 370) = 8.91, *p* = 0.01. The most commonly observed form of aggression was physical (*N* = 252, 68.1%), followed by verbal (*N* = 72, 19.5%) and relational (*N* = 46, 12.4%).

### 3.1. Sex Differences in Aggression and Victimisation

To examine whether there were sex differences in the number of times children were observed displaying the three different types of aggression when the FC was observed behaving aggressively, a multivariate analysis of variance (MANOVA) was conducted. This was not significant, Wilks’ *λ* = 0.94, *F* (3,52) = 1.08, *p* = 0.37, suggesting that there was no significant difference in the level of aggression displayed by boys and girls.

In order to examine whether boys and girls were more likely to use different forms of aggression, dichotomous variables were then created of whether the FC was ever observed behaving aggressively or not: overall; physically; verbally; and relationally. Chi-square analyses indicated no significant association between sex and overall likelihood of being observed behaving aggressively, *χ*^2^ (1*df*, *N* = 56) = 0.90, *p* = 0.34. However, there was a significant association for physical aggression, with more boys than girls ever being observed being physically aggressive, *χ*^2^ (1*df*, *N* = 56) = 4.18, *p* = 0.04, *V* = 0.27 (boys, *N* = 26, 76.5%; girls, *N* = 11, 50.0%). There was no significant association by sex for being verbally aggressive, *χ*^2^ (1*df*, *N* = 56) = 0.39, *p* = 0.53 or relationally aggressive, *χ*^2^ (1*df*, *N* = 56) = 1.08, *p* = 0.30.

Similar analyses were carried out for when the FC was observed as the target of another child’s aggression (victimisation). The MANOVA carried out on the numbers of times (as a proportion of the total number of times they were observed) boys and girls experienced victimisation: overall; physical victimisation; verbal victimisation; and relational victimisation was not significant, Wilks’ *λ* = 0.99, *F* (3,52) = 0.11, *p* = 0.96. Dichotomous variables were then created of whether the FC was ever observed being victimised or not: overall; physically; verbally; and relationally. Chi-square analyses were not significant for victimisation: overall, *χ*^2^ (1*df*, *N* = 56) = 0.12, *p* = 0.73; physical, χ^2^ (1*df*, *N* = 56) = 0.62, *p* = 0.43; verbal, *χ*^2^ (1*df*, *N* = 56) = 0.10, *p* = 0.75; relational, *χ*^2^ (1*df*, *N* = 56) = 0.47, *p* = 0.49.

#### Sex of Aggressors and Targets

For each observation of the FC behaving aggressively, the sex of the target of their aggression was noted. Across the observations where the FC was observed as behaving aggressively, their aggression was categorised as either same-sex only, other-sex only or both-sexes. For the targets of the FC’s aggression, there were 16 (38.1%) who had same-sex only targets, 4 (9.5%) other-sex only targets and 22 (52.4%) both-sexes targets across the observations. Chi-square analysis indicated that children were least likely to be aggressive towards children of the other sex only, *χ*^2^ (2*df*, *N* = 42) = 12.00, *p* = 0.002. For incidents where the FC was the victim, there were 25 (53.2%) FC who had same-sex aggressors only, 10 (21.3%) were victims of other-sex aggressors only and 12 (25.5%) were victimised by aggressors of both sexes. Chi-square analysis indicated that children were least likely to be victimised by children of the other sex only and most likely to be victimised by children of the same sex as them, *χ*^2^ (2*df*, *N* = 47) = 8.47, *p* = 0.014.

### 3.2. Behaviour of Onlookers

#### 3.2.1. Behaviour of FC as an Onlooker

The behaviour of the FC as an onlooker of aggression and victimisation was examined, see Table 2a. There were 114 incidents when the FC observed aggression by others: physical (*N* = 76, 66.7%), verbal (*N* = 29, 25.4%) and relational (*N* = 9, 7.9%). In the great majority of incidents in which the FC witnessed aggression and victimisation (*N* = 98, 86.0%), they did nothing (as observed during the observation period). In relatively few incidents (*N* = 14, 12.3%), they helped the victim (defender); and only very rarely (*N* = 2, 1.8%) did they help the aggressor (assistant). There were no observations of the FC encouraging the aggressor (reinforcer).

Due to small *N*s in observations of helping the victim and helping the aggressor, these were collapsed into an “active” behaviour category. A paired *t*-test indicated that children were more likely to do nothing when observing aggression or victimisation than to “actively” respond, *t* (41) = 10.32, *p* < 0.001, *d* = 1.59, 95% CI (0.62, 0.92).

There were sex differences in the ways in which FC responded to observations. Boys were more likely than girls to have ever been observed to take a “passive” (doing nothing) role when observing aggression *χ*^2^ (1*df, N* = 56) = 5.06, *p* = 0.024, *φ* = − 0.30; 82.4% (*N* = 28) of boys responded by doing nothing, compared with 54.5% (*N* = 12) of girls. There was no significant difference in whether boys or girls ever demonstrated an “active” (defending or assisting) response, *χ*^2^ (1*df*, *N* = 56) = 0.003, *p* = 0.61 (Fisher’s Exact performed); 17.6% (*N* = 6) of boys and 18.2% (*N* = 4) of girls had at least one active response when observing aggression.

Due to the relatively small *N*s, it was not possible to examine statistically whether the FC was more likely to take an “active” (defending or assisting) or “passive” (doing nothing) role when observing the different forms of aggression. However, a trend in the data suggested that they were more likely to be observed taking an “active” role when observing relational aggression by others than either physical or verbal aggression (see Table 2).

#### 3.2.2. Behaviour of Onlookers when FC Was Being Aggressive or Victimised

The behaviour of others as an onlooker of aggression or victimisation experienced by the FC was examined, see Table 2b. There were 262 incidents of the FC being either aggressive or victimised: physical (*N* = 176, 67.2%); verbal (*N* = 43, 16.4%); and relational (*N* = 37, 14.1%) (*N* = 6, 2.3% were uncoded). In nearly two thirds of these cases (*N* = 164, 62.6%), there was another child/other children present.

When the behaviour of onlookers was examined, there were 173 recorded onlooker behaviours across the incidents of the FC being aggressive or victimised. Of these behaviours, the majority were doing nothing (*N* = 121, 69.9%) and the remaining onlookers were helping the aggressor (*N* = 31,17.9%) or helping the victim (*N* = 17, 9.83%). The fewest were observed encouraging the aggressor (*N* = 4, 2.3%).

A mean of 1.13 children (ranging from 0 to 8) were present when the FC was aggressive or victimised. The number of children observed doing nothing when present during episodes of aggression/victimisation was highest (mean per incident = 0.77, *SD* = 1.04), followed by helping the aggressor (mean = 0.21, *SD* = 0.63), helping the victim (mean = 0.10, *SD* = 0.34) and with fewest encouraging the aggressor (mean = 0.06, *SD* = 0.32).

Chi-square analysis was performed to examine whether there was a significant association between type of aggression/victimisation and likelihood of there being another child present or not. There was no significant association, *χ*^2^ (2*df*, *N* = 254) = 5.26, *p* = 0.07. A one-way ANOVA indicated that there was no significant effect of aggression/victimisation type on the number of children present, *F* (2, 251) = 1.74, *p* = 0.18.

Chi-square analysis indicated that there was more likely to be at least one “active” onlooker (classified as helping the aggressor, helping the victim or encouraging the aggressor) during episodes of relational aggression/victimisation than when physical or verbal aggression/victimisation was taking place, *χ*^2^ (2*df*, *N* = 254) = 9.24, *p* = 0.01, *V* = 0.19. In nearly a third of observations of relational aggression/victimisation (*N* = 11, 30.6%), onlookers were observed taking an “active” role, in comparison to physical (*N* = 31, 17.7%) and verbal (*N* = 2, 4.7%). It was not possible to examine sex differences in onlooker behaviour when the FC was aggressive or victimised as onlooker sex was not recorded.

## 4. Discussion

This study observed physical, verbal and relational aggression in young children aged 4–5 years, with physical aggression being the most common, in line with previous studies [4]. There were more observations where the FC was being aggressive than when they were being victimised or observing aggression/victimisation. It is unclear why this might be and warrants further investigation. When the focal child was victimised, they were more likely to be victimised by children of the same sex to them than by children of the other sex or by both sexes. When the focal child was behaving aggressively, they were also more often observed behaving aggressively to children of the same sex to them or to both boys and girls, rather than solely to children of the other sex. This ties in with previous observational studies [22], which found that boys were more likely to behave aggressively towards other boys, and when girls were aggressive, they targeted other girls. It also partially confirms the results of social network analysis of aggressive behaviour [23] where boy–boy and girl–boy dyads of victimisation were more common than girl–girl or boy–girl dyads. This could indicate that children this age are being more specific in their choice of victim than Monks and colleagues [8,26] might have suggested. However, the increased likelihood of children targeting others who are of the same sex as them may not indicate true specificity in their choice of target, but perhaps just opportunity. Children’s peer groups are relatively sex-segregated during early childhood [56], so it is possible that children behave aggressively towards those children with whom they socialise more frequently. In support of this, Hanish et al. [57] found that when they controlled for the degree to which children were segregated by sex, the levels of aggression towards same-sex or other-sex classmates were not significantly different.

The main objective of this study was to examine whether assisting, reinforcing and defending behaviours could be identified during episodes of peer victimisation among a sample of young children. This observational study found that children other than the aggressor and victim were frequently present during peer victimisation. When the focal child was being aggressive or victimised, there were other children present in nearly two thirds of occasions, with the number of “onlookers” present ranging from 0 to 8. On average, one child was present during these episodes of aggression or victimisation.

We found that in the majority of cases, onlookers were not observed doing anything related to the aggression/victimisation, similar to the findings of Vlachou et al. [44]. We have called this “doing nothing”. However, O’Connell et al. [31] described this as a form of reinforcing of the aggressor’s behaviour (it has some overlap with Salmivalli et al.’s [32] participant role of the reinforcer). O’Connell et al. [31] identified “peer passively joins in” where the child notices the bullying and does not leave the situation. This is similar to our category of “doing nothing”, although we did not note whether the child left the situation or remained there. This category of observation was originally aimed at exploring “outsider” behaviour, where the child does not do anything when they are aware of bullying [32]. We differentiated “outsider” from “reinforcer”, with a focus on reinforcing being more “active” with the child doing something (beyond observing) that reinforced the aggressor’s behaviour. However, we acknowledge that “doing nothing” could be viewed as a kind of “passive” reinforcing, by simply providing an audience that does not intervene.

Other children were “active” in less than a third of incidents of aggression by or victimisation of the FC; either helping the aggressor, encouraging the aggressor or defending the victim. Active reinforcement by encouraging the aggressor was particularly low in frequency of observation; either when the FC was aggressive or victimised or by the FC themselves. We did identify one sex difference in FC behaviour, with boys being more likely than girls to “do nothing”. There was no difference between boys and girls in “active” responses. Unfortunately, due to the sample size, it was not possible to examine the subtypes of active responses. It is possible that the responses by boys and girls behave in different “active” ways, as this category included both behaviour that was supportive of the aggressor and behaviour that was supportive of the victim.

There was no association between the type of aggression/victimisation and whether there was anyone else present. However, children were found to be more “active” when the aggression/victimisation was relational in nature than when it was physical or verbal. This was statistically significant for children observing aggression or victimisation of the FC and appeared as a trend in the data for the FC’s behaviour when observing the aggression/victimisation of others. It is not clear why this might be. Looking at the types of response, it may be because this form of aggression is easier to defend without putting oneself in the firing line of the aggressor. Defending could involve telling the excluded individual that they can come and play with you and your friends, rather than defending the aftermath of physical aggression or a verbal spat. Joining in when someone else has started the aggression may also be easier for a child when the aggression is relational in nature; it may feel less aggressive to concur with someone that another person cannot join in your game, compared to joining in physically or verbally abusing another. Furthermore, relational forms of aggression may by their nature be more likely to involve other parties, for example, excluding someone from a group activity or gossiping. Although research with older children suggested that onlookers might be more likely to intervene (defend) when they perceive the aggression to be more severe, in particular physical aggression [51], in the current study, it was not possible to disentangle different types of “active” responding by type of aggression, and so it may be that “active” responses for the different types of aggression may differ.

A limitation to this study was the small sample size, although the detailed observations which totalled 2610 min enabled examination of some of the behaviours children displayed during episodes of peer victimisation. In addition, although each observation period was 10 min in duration, it was not possible to observe what happened afterwards. A child who was observed “doing nothing” may have later comforted the child being victimised or told an adult what had happened. If this occurred after the observation interval had terminated, then this would have been missed by the observer. Although we considered children to be “onlookers” if they were within 5 metres of the incident and were looking in that direction, it is still possible that some of our “onlookers” may not have been aware of aggression. Furthermore, it was not possible to examine sex differences in onlooking behaviour by the FC due to small *N*s, and in onlooking behaviour by others when the FC was behaving aggressively or being victimised, as recording the sex of onlookers was beyond the scope of this study. However, future studies should aim to examine this. Furthermore, it may be possible to examine whether there are same-sex groups of children who behave aggressively together. A further potential limitation was the use of field notes rather than video recordings of children’s behaviour. The use of video recordings would facilitate going back over observations to check coding. However, field notes were chosen within the current study as being more acceptable to schools and parents/carers.

This study focussed on children during their first year of compulsory schooling in the UK (aged 4–5 years). We argue that these findings suggest that peer victimisation is more dyadic and less of a group process than is identified among older groups. Although other children were observed as present during many episodes of peer victimisation, they were most commonly passive onlookers, rather than taking on participant roles as identified among older groups. Future research could examine the development of these behaviours during the formative school years to explore how and at what point the peripheral roles of assistant and reinforcer become more clearly observable and frequent. These developmental changes may be related to social and cognitive developments during childhood, such as social understanding [4].

The findings from this observational study concur with child reports of peer victimisation which suggest that peer victimisation is less group-led at that age. This indicates that child reports reflect the dyadic nature of peer victimisation during early childhood, rather than this being an artefact of children’s cognitive limitations. This has implications for the methodological approaches we use to research peer victimisation during early childhood. We propose that these findings suggest that peer reports may be a useful tool to use with young children when investigating peer victimisation.

Peer victimisation among children during the preschool or early school years seems to differ qualitatively from what is observed among older children. Prevention and intervention work against aggression and victimisation needs to be tailored to meet the needs of younger children [58]. For example, work with children on how to respond when they observe peer victimisation may be especially relevant. However, it is important to recognise that this would need to be part of a larger-scale project, so that children are supported in this. An example of such an approach may be the Free of Bullying programme launched in Denmark in 2007 [59]. The philosophy of the programme is to consider bullying as a group phenomenon, and to foster four fundamental values in the classroom: tolerance, respect, care and courage. Some evaluation based on interviews with teachers and children concluded that the children in preschools and primary schools where the intervention was delivered were more equipped to deal with bullying.

## 5. Conclusions

In summary, the current study found that children other than the aggressor and victim were present in nearly two thirds of the observations of peer victimisation. However, most of these children were observed “doing nothing” rather than defending the victim or joining in with the aggression. The category “doing nothing” indicates that there is often an audience for the aggression, which may, as O’Connell et al. [31] suggested, provide passive reinforcement for the aggressor.

Nevertheless, our findings support earlier studies from peer and teacher reports which have mainly found that the more peripheral participant roles are poorly defined among this age group [9,26]. Furthermore, it supports the hypothesis that such findings found from peer nominations are due to actual behavioural differences, rather than just cognitive limitations in reporting. Peer victimisation among younger children appears to be less group-led than is found among middle childhood and adolescence [32,38,39].

## Figures and Tables

**Table 1:** Number of times each type of aggression was observed by the focal child (FC) role.

Role of FC	Physical	Verbal	Relational	Total
Aggressor	104	26	20	150
Victim	72	17	17	106
Observer	76	29	9	114
Total	252	72	46	370

For 6 observations of FC aggression/victimisation, the type of behaviour was uncoded.

**Table 2:** Behaviour of onlookers during episodes of aggression/victimisation (number of incidents and percentage of total).

**(a) Behaviour of Focal Child (FC) When Observing Aggression by Others, by Aggression Type**				
**Aggression Type**	**Did Nothing**	**Helped** **Victim**	**Helped** **Aggressor**	**Encouraged** **Aggressor**
Physical	68 (89.5%)	7 (9.2%)	1 (1.3%)	-
Verbal	24 (82.8%)	5 (17.2%)	0 (0.0%)	-
Relational	6 (66.7%)	2 (22.2%)	1 (11.1%)	-
Total	98 (86.0%)	14 (12.3%)	2 (1.8%)	-
**(b) Behaviour of Onlookers When Focal Child (FC) Observed Being Aggressive/Victimised by Behaviour Type**				
**Aggression/Victimisation Type**	**Did Nothing**	**Helped** **Victim**	**Helped** **Aggressor**	**Encouraged** **Aggressor**
Physical	81 (69.2%)	12 (10.3%)	21 (18.0%)	3 (2.6%)
Verbal	21 (91.3%)	1 (4.4%)	1 (4.4%)	0 (0.0%)
Relational	19 (57.6%)	4 (12.1%)	9 (27.3%)	1 (3.0%)
Total ^1^	121 (69.9%)	17 (9.8%)	31(17.9%)	4 (2.3%)

^1^ Note that more than one onlooker behaviour could be recorded per incident.

## Data Availability

The data presented in this study are available on request from the corresponding author. The data are not publicly available due to ethical requirements.
